# Expression of Glucagon-Like Peptide-1 Receptors in the Submandibular Gland of Mice and Its Implications in Type 2 Diabetes

**DOI:** 10.7759/cureus.70465

**Published:** 2024-09-29

**Authors:** Masato Akakura, Ippei Watari, Minami Watanabe, Srisutha Jiratchaya, Takashi Ono

**Affiliations:** 1 Department of Orthodontic Science, Graduate School of Medical and Dental Sciences, Tokyo Medical and Dental University (TMDU), Tokyo, JPN; 2 Department of Orthodontics, Faculty of Dentistry, Chulalongkorn University, Bangkok, THA

**Keywords:** glp-1 receptor, high fat diet, incretin, streptozotocin, submandibular gland, type 2 diabetes

## Abstract

Introduction

Type 2 diabetes mellitus (T2DM) not only affects the pancreas directly involved in glucose metabolism but also impairs salivary gland function. Glucagon-like peptide-1 (GLP-1) is a gastrointestinal hormone that lowers postprandial blood glucose levels by stimulating insulin secretion from the pancreas. Previous studies have revealed the presence of GLP-1 receptors (GLP-1R) in salivary glands. However, the effect of diabetes on salivary gland GLP-1R remains unclear. This study aimed to observe the impact of T2DM on GLP-1R in the submandibular gland (SMG).

Materials and methods

Twenty-five-week-old mice were randomly divided into four groups (n=5 each): 11w and 13w control groups (CON), and 11w and 13w diabetes mellitus groups (DM). After a five-day adaptation period, the DM group mice were subjected to a high-fat diet, while the CON group received a standard diet. The DM group mice were then induced into a state of T2DM by a single low-dose streptozotocin injection at nine weeks of age. Oral glucose tolerance tests (OGTT) were conducted to evaluate mouse glucose tolerance. At 11 and 13 weeks of age, SMG was excised under general anesthesia, and the morphology of SMG was evaluated by hematoxylin-eosin staining, while the distribution and expression of GLP-1R were assessed by immunohistochemical staining. The data obtained were subjected to the Shapiro-Wilk test to confirm normal distribution, the t-test for the OGTT results, and statistical analysis for other results by one-way analysis of variance.

Results

Consistent with previous reports, the mice in the DM group showed higher body weight and lower glucose tolerance. Histological analysis revealed an increase in the acinar area and a decrease in the ductal area of the SMG in the DM group. Although there was no significant decrease in the cell count regarding the ductal area, a tendency toward luminal dilation was observed. Interestingly, the expression pattern of GLP-1R was limited to the ductal portion of the SMG, with a decrease in anti-GLP-1R-positive areas observed in the DM group compared to the CON group. While there was no significant difference in anti-GLP-1R-positive areas between the CON11w and CON13w groups, the DM13w group exhibited a significant decrease compared to the DM11w group. These data suggest that diabetes induces both structural changes in the SMG and a reduction in GLP-1R expression, particularly in the ductal regions.

Conclusions

We found that the expression level of GLP-1R in SMG was decreased in the DM group mice. This data demonstrates the potential relationship between T2DM and GLP-1R expression. Moreover, there was an indication of a temporal decrease in anti-GLP-1R positive areas over time. This result may suggest the involvement of salivary gland GLP-1R in the mechanism of impaired SMG function caused by T2DM, potentially mediated through the decrease in blood GLP-1 levels.

## Introduction

The progression of diabetes increases the risk of multiple complications and causes organ morbidity in the body. Particularly in the oral cavity, xerostomia is known as a symptom of diabetes. The salivary glands primarily consist of three major glands: the submandibular gland (SMG), the parotid gland (PG), which is the largest, the sublingual gland (SLG), and the minor salivary glands. Saliva serves multiple functions, including maintaining the moist state of the oral mucosa, perceiving taste and smell, initiating digestion at the initial stage, and protecting teeth [1]. Therefore, various receptors are involved in salivary secretion, with α-adrenergic receptors and muscarinic receptors promoting both fluid secretion and ductal secretion [2,3]. The salivary glands exert multiple functions through the expression and action of various receptors.

In the rat major salivary gland, incretin and its receptors have been reported to be expressed in the ductal portion [4,5]. Incretin is a gastrointestinal hormone that acts on pancreatic β-cells to stimulate insulin secretion, with two types such as glucagon-like peptide 1 (GLP-1) and glucose-dependent insulinotropic polypeptide (GIP). Upon stimulation by food passing through the digestive tract, GIP is secreted from K cells in the upper small intestine, while GLP-1 is secreted from L cells in the lower small intestine. Incretin regulates insulin secretion from pancreatic β cells, promoting a decrease in blood sugar levels. It inhibits glucagon secretion, suppresses the feeding center and inhibits gastric motility and gastric acid secretion. The expression of incretin and its receptors has also been observed in the myocardial cells, brain, vascular endothelial cells, adipocytes, osteoblasts, and liver [6]. GLP-1 receptors (GLP-1R) are expressed in the intragemmal nerves adjacent to the taste cells in the mouse taste buds, and GLP-1R knockout mice show a reduced taste response to sweetness [7]. It is possible that GLP-1R plays a role in some of the physiological functions within the oral cavity.

GIP and GLP-1 possess distinct biological characteristics. Although both GIP and GLP-1 stimulate insulin secretion, their effects differ in type 2 diabetes mellitus (T2DM). In type 1 diabetes mellitus (T1DM), the destruction of pancreatic beta cells leads to a lack of insulin production. Unlike T2DM, where insulin resistance and beta cell dysfunction coexist, the secretion of both GIP and GLP-1 is generally preserved in T1DM, although their effects might be blunted due to the absence of insulin-producing cells. In T2DM patients, GLP-1 secretion is impaired, while GIP secretion is unaffected [8]. Moreover, it has been reported that in T2DM patients, GIP administration has little effect on stimulating insulin secretion, whereas GLP-1 administration activates glucose-dependent insulin secretion. This suggests that the impact that is received and exerted by T2DM is stronger for GLP-1 than for GIP, and attention has been focused on the function of GLP-1 in glucose metabolism.

T2DM induces functional damage in various organ systems such as the heart, blood vessels, kidneys, and nervous system, causing salivary gland dysfunction characterized by reduced saliva secretion [9]. It has also been reported that uncontrolled diabetic patients exhibit higher salivary glucose levels compared to non-diabetic individuals [10]. There are also reports of the effects of T2DM on salivary glands in animal models. In SMGs of db/db mice, which develop T2DM due to leptin receptor deficiency, histological changes such as an increase in acinar area and a decrease in ductal area have been observed [11].

T2DM induces an aforementioned decline in salivary gland function, supported by reported histological changes. This suggests that T2DM may also impact the salivary gland GLP-1R. However, there have been no reports on the effects of T2DM on salivary gland GLP-1R. Therefore, the aim of this study was to investigate an altered salivary gland GLP-1R expression due to T2DM. In this study, the working hypothesis was that T2DM leads to a change in SMG GLP-1R expression using a T2DM mouse model induced by a high-fat diet (HFD) intake and streptozotocin (STZ) administration.

## Materials and methods

Animals and experimental design

The animal protocol was approved by the Institutional Animal Care and Use Committee of Tokyo Medical and Dental University (A2022-090A), and experiments were carried out under the control of the institutional guidelines for animal experiments.

Twenty-five-week-old male C57Bl/6J mice purchased from Sankyo Labo Service (Tokyo, Japan) were used for the experiment. After five days of adaptation, the mice were randomly divided into four groups (n=5 each), the control (CON) group (CON11w and CON13w), and the type 2 diabetic mellitus (DM) group (DM11w and DM13w). The CON11w and DM11w groups were sacrificed at 11 weeks of age, while the CON13w and DM13w groups were sacrificed at 13 weeks of age. The CON mice were fed a standard diet (CE-2; CLEA, Tokyo, Japan) with a total energy of 3.402 kcal/g and 4.6% fat kcal. The DM mice were fed an HFD (HFD32; CLEA, Tokyo, Japan) with a total energy of 5.076 kcal/g and 32% fat kcal. After feeding HFD for three weeks, mice in both the DM11w and DM13w groups were fasted for four hours. The DM mice were injected intraperitoneally with a low dose (40 mg/kg) of STZ (Sigma-Aldrich, St. Louis, USA) diluted in 0.05 M citrate buffer (pH 4.5), and the CON mice were injected intraperitoneally with vehicle (citrate buffer) only. This method follows the National Institutes of Health's (NIH) Diabetic Complications Consortium protocol [12].

**Figure 1:**
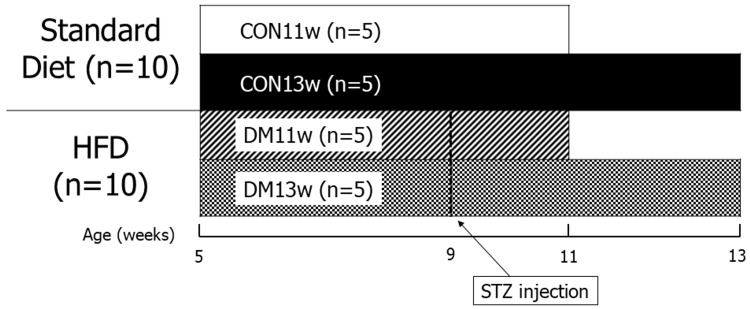
Experimental design The control group was fed a regular diet, while the DM group was fed a high-fat diet. At nine weeks of age, low-dose streptozotocin was administered to induce type 2 diabetes mellitus status in the diabetes mellitus group. Animals were sacrificed at 11 weeks (CON11w and DM11w) and 13 weeks (CON13w and DM13w) of age, and samples were collected for analysis. HFD: high-fat diet; CON: control group; DM: diabetes mellitus; STZ: streptozotocin

Throughout the experiment, food consumption was monitored daily and body weight was monitored twice weekly. The oral glucose tolerance test (OGTT) was performed weekly after STZ administration. Mice were euthanized at six and eight weeks after starting the experiment, i.e., at 11 and 13 weeks of age, and the SMG was immediately collected and analyzed using HE staining and immunohistochemistry. A graphical summary of the experimental flow is shown in Figure 1. The temperature, humidity, and lighting (12-hour light/dark cycle) were controlled under identical environmental settings, and the animals were allowed to consume water ad libitum throughout the experiment.

OGTT

OGTTs were performed at 11 and 13 weeks of age after STZ administration to confirm that mice could be induced to the T2DM state. The two DM group mice were fasted overnight for 18 hours before the test. Blood glucose levels were first measured at 0 minutes with blood samples collected from the tail picked. D-(+)-glucose (Nakalai Tesque, Kyoto, Japan) was adjusted to a dose of 1 g/kg body weight and administered orally at a volume of 10 ml/g body weight. Blood glucose measurements were performed at 30, 60, 90, and 120 minutes as well as at 0 minutes using the ACCU-CHEK Guide blood glucose measurement kit (Roche Diagnostic, Basel, Switzerland). The area under the curve (AUC) was calculated to evaluate blood glucose levels.

Histological preparation of SMG

Collected SMGs were immediately immersed in 4% paraformaldehyde (MildformÒ, Wako Pure Chemical Industries, Osaka, Japan) at 4°C for 24 hours and then embedded in paraffin wax. After fixation, the tissues were washed with PBS solution at pH 7.4 and paraffin-embedded according to the customary method. The paraffin-embedded samples were thinned to 5 μm using a microtome (Leica Biosystems, Nussloch, Germany), and serial tissue sections were prepared [13].

Hematoxylin-eosin (HE) staining

For HE staining, the samples were first deparaffinized three times in xylene, three times in 100% ethanol, and then continuously in 90%, 80%, and 70% ethanol. The samples were then rinsed in running water. All procedures were performed in three minutes. Then the samples were stained with hematoxylin for 12 minutes and washed with running water for 10 minutes. After counterstaining with eosin for one minute and 15 seconds, the samples were rinsed with 90% and 100% ethanol and dehydrated with xylene for one minute each. Finally, the samples were sealed with Mountquick (Daido Sangyo, Toda, Japan). Histological features of SMGs were observed under a light microscope (Microphoto-FXA; Nikon, Tokyo, Japan) at 400x magnification. Images were captured with a digital camera (DXm1200; Nikon, Tokyo, Japan). For histological evaluation, image analysis software (Image J 1.53f51, NIH, Maryland, USA) was used for the following items: percentage of ductal and acinar area, number of cells in the duct, ductal cell area, percentage of parenchymal ductal area under 400x magnification. The percentage of the ductal and acinar area was calculated by measuring the duct area excluding the lumen in one field of view (170 x 210 µm) and subtracting it from the one field of view area (excluding the lumen). The number of duct cells was counted in an area of 0.5 x 0.5 mm. The duct cell area was calculated by finding the duct area in the area and dividing it by the number of cells. The parenchymal duct area was calculated by dividing the duct area including the lumen by the duct area without the lumen.

Immunohistochemical staining and histological evaluation of GLP-1R

To evaluate the expression and distribution of GLP-1R in SMG, immunohistochemical staining was performed using an anti-GLP-1R antibody. This method was adapted based on the approach used by Pornchanok Sangsuriyothai in previous research [14]. Sections were first deparaffinized with xylene and rehydrated with ethanol. Then, the inactivation of endogenous peroxidase was performed by immersion in 3% hydrogen peroxide solution for 15 minutes. After washing with tris-buffered saline with Tween-20 (TBST), sections were acted on overnight at 4°C with anti-rabbit polyclonal GLP-1R antibody (#218532, 1:500, Abcam, Cambridge, USA) diluted in PBS containing 0.1% BSA. After the reaction, the sections were washed with TBST for three minutes, and the following operations were performed using the VECTASTAIN Elite ABC-HRP Kit (PK-6101, Vector Laboratories, North Brunswick, USA). First, sections were incubated with anti-rabbit IgG secondary antibody of the ABC kit for 30 minutes at room temperature and then washed three times with TBST for three minutes. Finally, the VECTASTAIN Elite ABC Kit reagent was applied for 30 minutes and washed three times with TBST for three minutes. The sections were then treated with 3,3-diaminobenzidine (DAB substrate kit; #ab64238, Abcam, Cambridge, USA) for 10 minutes and washed twice with distilled water for five minutes. The tissues were then contrast-stained with Mayer's hematoxylin (Fujifilm Wako Pure Chemical Corp., Osaka, Japan) and washed in running water for 10 minutes. After washing, tissues were dehydrated in a graded alcohol series and xylene and sealed with Mount-Quick (Daido Sangyo, Toda, Japan).

For evaluation of GLP-1R localization changes in SMG, anti-GLP-IR immune-positive areas were observed under a light microscope (Nikon ECLIPSE80i, Nikon, Tokyo, Japan). Two sections per sample and one section per six fields of view of 300 × 300 pixels (0.05 x 0.05 mm) were observed at 400x magnification. All procedures for image capture, identification, and processing were standardized before images were captured. Six fields of view for each section were randomly selected. Positive immunohistochemical regions were extracted using a digital image analyzer (Image J 1.53f51, NIH, Maryland, USA), and the immune intensity in each image was measured as optical density (OD).

Statistical analysis

Statistical analysis was performed using GraphPad Prism 9 (GraphPad Software Inc., La Jolla, USA). All data are presented as mean ± standard error of the mean (SEM), and the normal distribution was evaluated using the Shapiro-Wilk test. Statistical significance was defined as p<0.05. An unresponsive Student's t-test was applied to compare body weights, OGTT values, and AUC data for both groups of mice at 11 and 13 weeks of age. For comparisons of the percentage of acinar and ductal area, number of duct cells, duct cell area, parenchymal duct area, and anti-GLP-1R immunopositive area compared between the four groups (CON11w, DM11w, CON13w, and DM13w), a one-way analysis of variance (ANOVA) followed by the Holm-Sidak multiple comparisons test was used.

## Results

Effect on mice body weight

**Figure 2:**
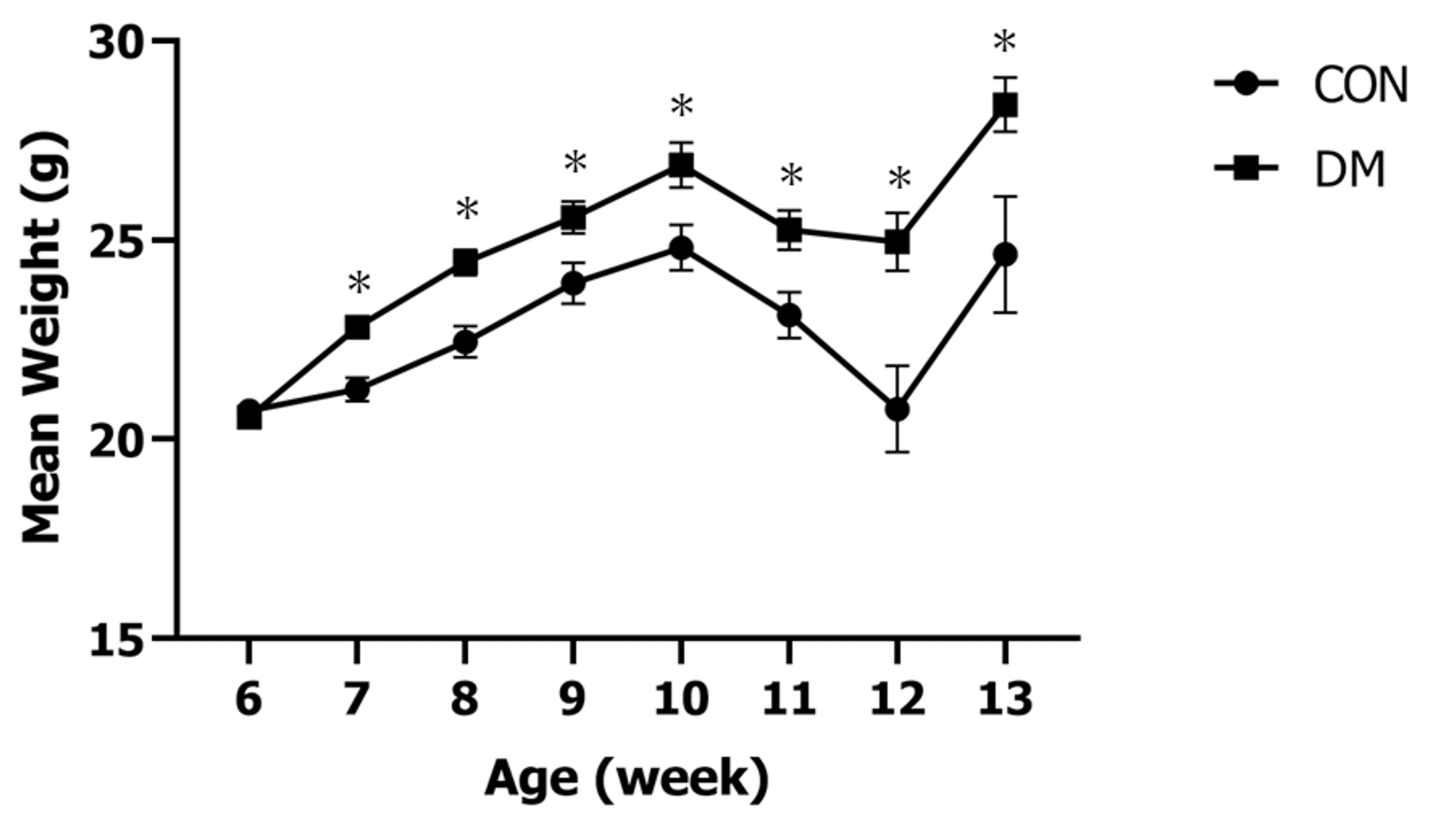
Body weight changes in the control and experimental animals ^*^p<0.05 CON: control group; DM: diabetes mellitus

Body weights at the beginning of the experiment did not differ significantly among the groups. Mice in the DM group began to gain weight the week after the HFD was given; at seven weeks of age, the average body weight increased by 1.52 g. Thereafter, the T2DM group continued to gain predominantly over the CON group until 11 and 13 [正赤1] [正赤2] weeks of age (Figure 2).

Effect on mice glucose tolerance

**Figure 3:**
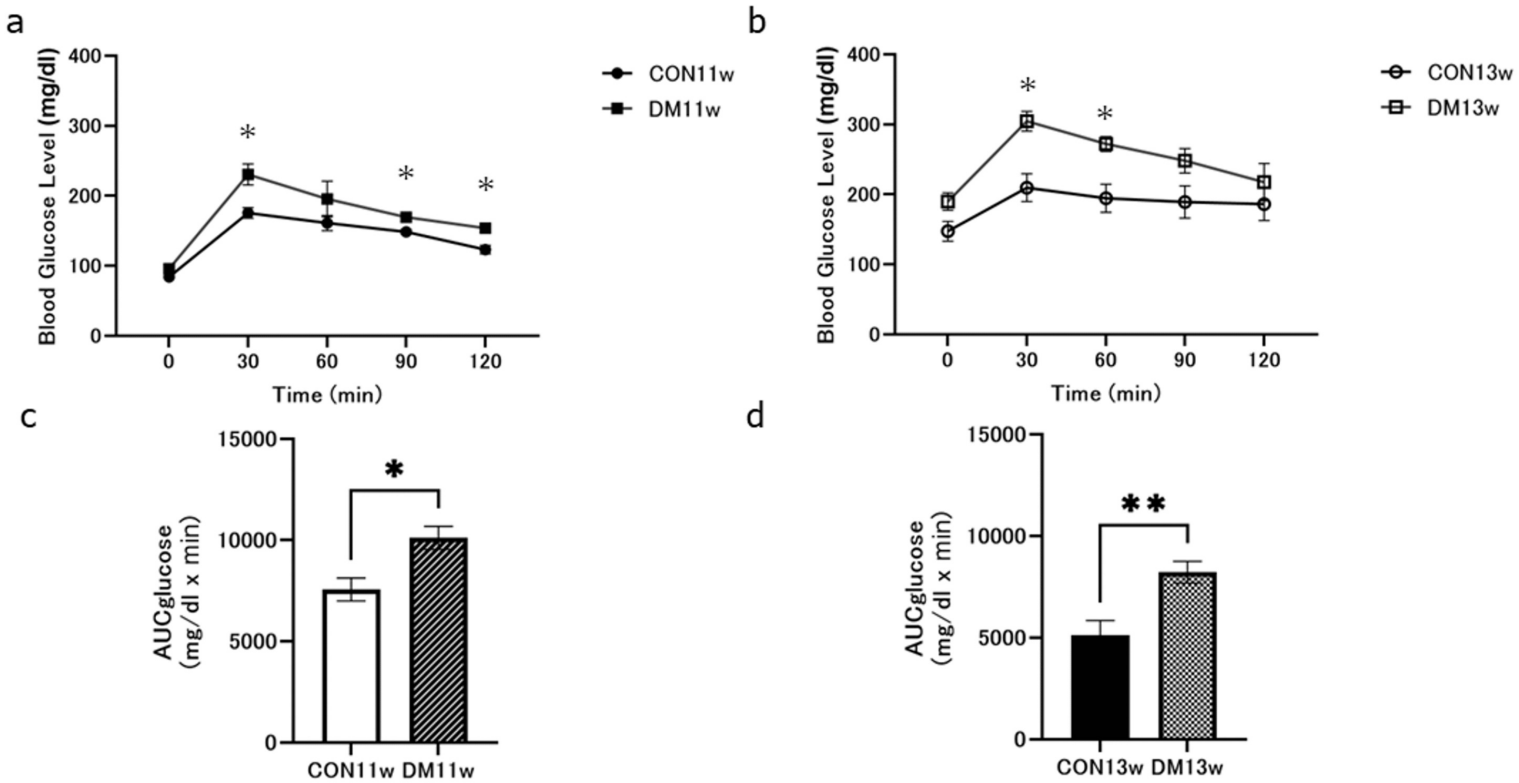
OGTT examination and AUC in the control and experimental groups a) Comparison of OGTT between the CON and DM groups at 11 weeks; b) Comparison of OGTT between the CON and DM groups at 13w; c) Comparison of AUC of OGTT at 11 weeks; d) Comparison of AUC of OGTT at 13w. ^*^p<0.05; ^**^p<0.01 CON: control group; DM: diabetes mellitus; OGTT: oral glucose tolerance test; AUC: area under the curve

Before euthanasia, glucose tolerance was assessed by performing an OGTT on the mice to confirm that they were in T2DM status. OGTTs were performed in all groups, and AUCs were calculated; for the 11w and 13w OGTTs, all mice had peak blood glucose levels at 30 minutes, which then decreased until 120 minutes. For both tests, blood glucose levels in the DM group were significantly higher than those in the CON group at 30 minutes. The DM group also showed significantly larger OGTT values at 90 minutes and 120 minutes at 11 weeks (Figure 3a). On the other hand, the OGTT value at 13 weeks was significantly larger in the DM group at 60 minutes (Figure 3b). The AUC of the DM groups was significantly larger than that of the CON11w and CON13w, respectively (Figures 3c-3d).

Morphological changes in mice SMG

**Figure 4:**
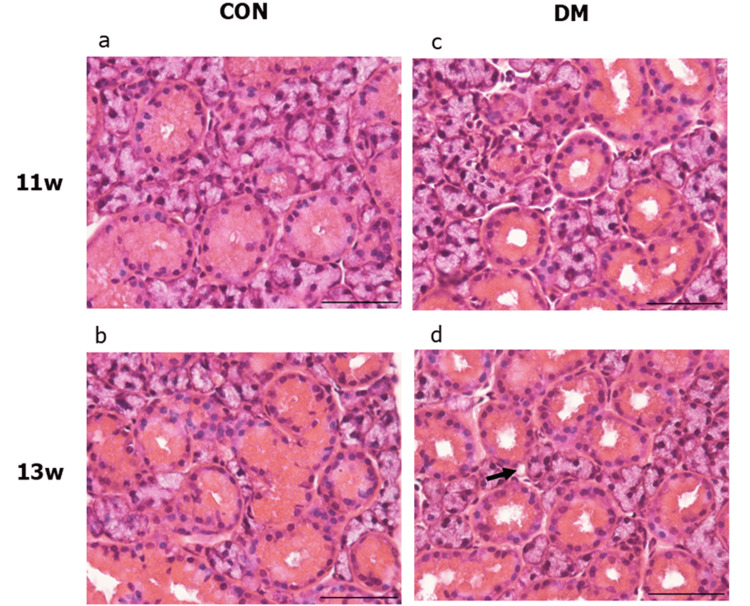
Hematoxylin-eosin staining of the SMG Morphological observation of the CON group at 11 weeks (a) and 13 weeks (b), and the DM group at 11 weeks (c) and 13 weeks (d). DM group showed an enlargement of the glandular atrial cells and an expansion of the luminal area. The DM13w group showed vacuolation of atrial cells (arrow in d). The scale bar represents 50 µm. a~d: magnification: 400x CON: control group; DM: diabetes mellitus; SMG: submandibular gland

**Figure 5:**
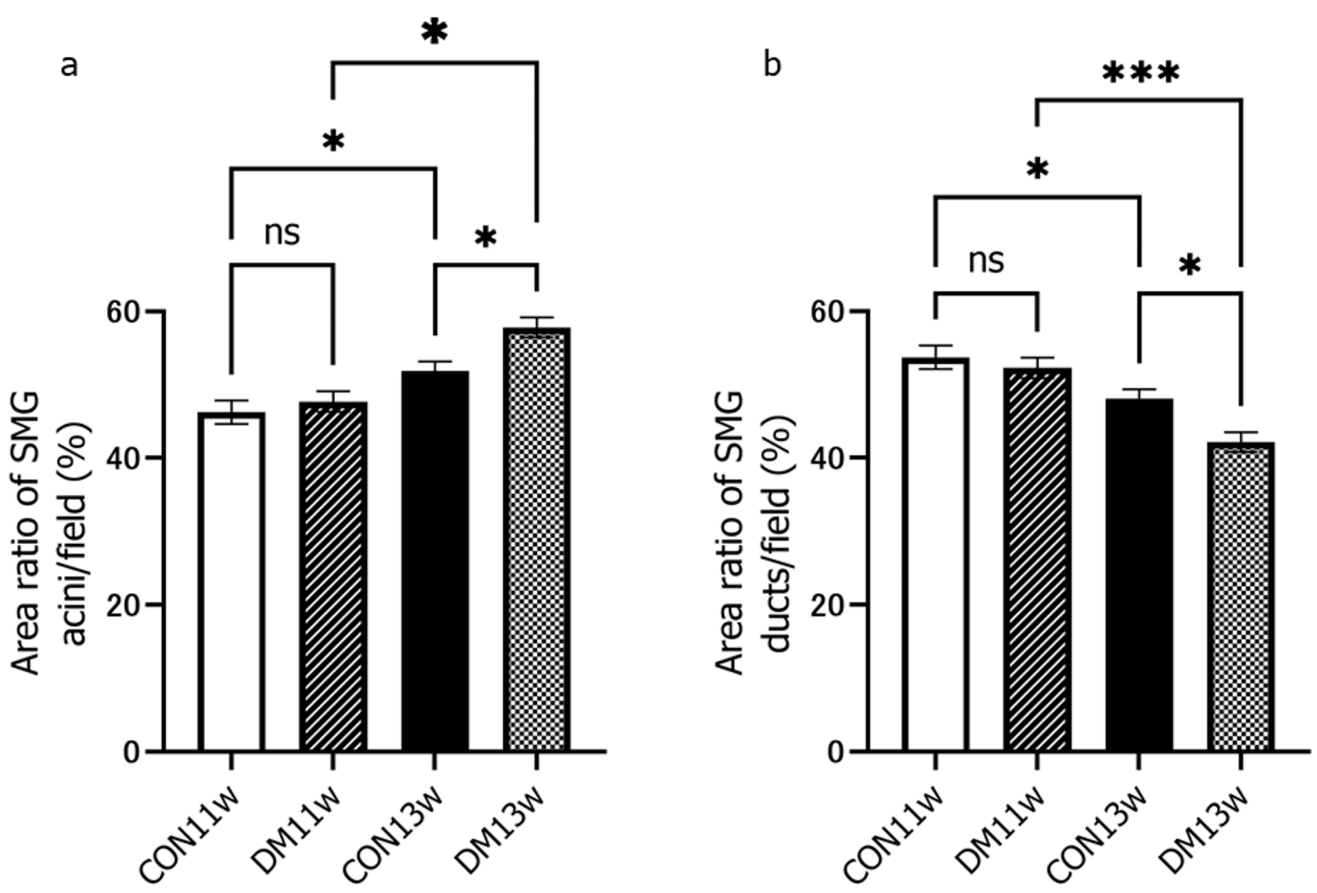
Percentage of acinar areas and percentage of duct areas The percentage of acinar (a) and ductal (b) area was calculated by measuring the duct area excluding the lumen in one field of view (170 x 210 µm) and subtracting it from the one field of view area (excluding the lumen). ^*^p<0.05;^ ***^p<0.001 CON: control group; DM: diabetes mellitus; ns: not significant

**Figure 6:**
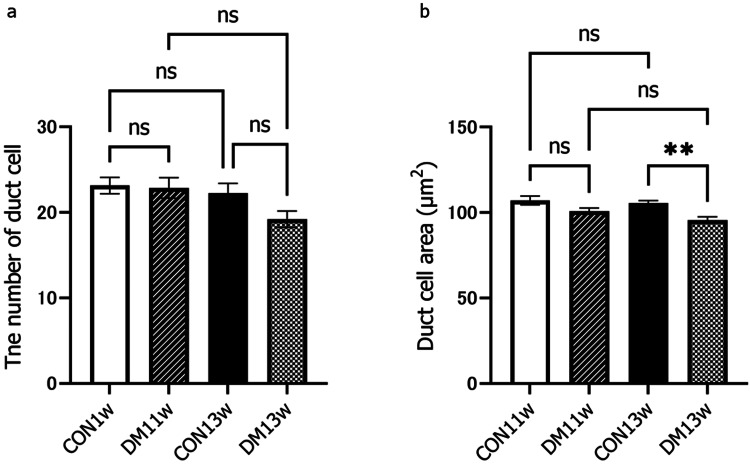
Number of duct cells and duct cell area The number of duct cells (a) was counted in an area of 0.5 x 0.5 mm. Duct cell area (b) was calculated by finding the duct area in the area and dividing it by the number of cells. ^**^p<0.01 CON: control group; DM: diabetes mellitus; ns: not significant

**Figure 7:**
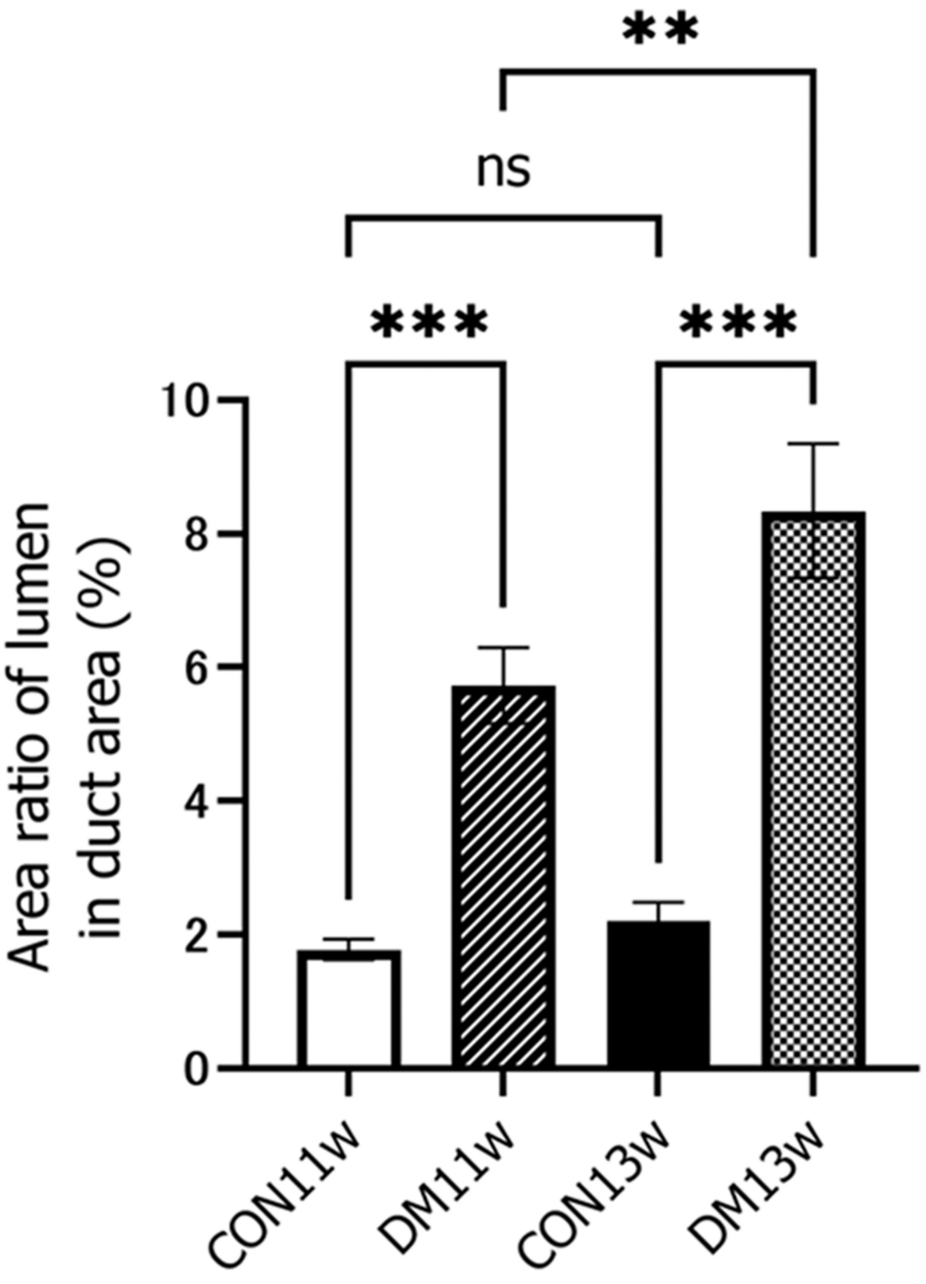
Area ratio of lumen in the duct area The area ratio of the lumen in the duct area was calculated by dividing the duct area including the lumen by the duct area without the lumen. ^**^p<0.01; ^***^p<0.001 CON: control group; DM: diabetes mellitus; ns: not significant

Under an optical microscope at 400x magnification, the CON group did not show any change over time in the acinar and duct cells (Figure 4), while the DM group showed enlargement of the acinar cells and luminal area in the duct at 11 and 13 weeks of age. Vacuolation of the acinar cell was also observed in the DM13w group (arrowhead). Morphometric analysis revealed that at 13 weeks, the area ratio of acini/field in the DM group increased significantly, while the area ratio of the duct decreased significantly (Figure 5). The number of duct cells per unit area did not change between the CON and DM groups or over time (Figure 6a). The area per duct cell was not significantly different between the CON and DM groups at 11 weeks of age but significantly decreased in the DM group at 13 weeks (Figure 6b). The area ratio of lumen in the duct was significantly greater in the DM group than in the CON group at both 11 and 13 weeks of age. The ratio of lumen in the DM group increased significantly from 11 to 13 weeks of age (Figure 7), while that in the CON group remained unchanged.

GLP-1R localization and expression in the SMG

**Figure 8:**
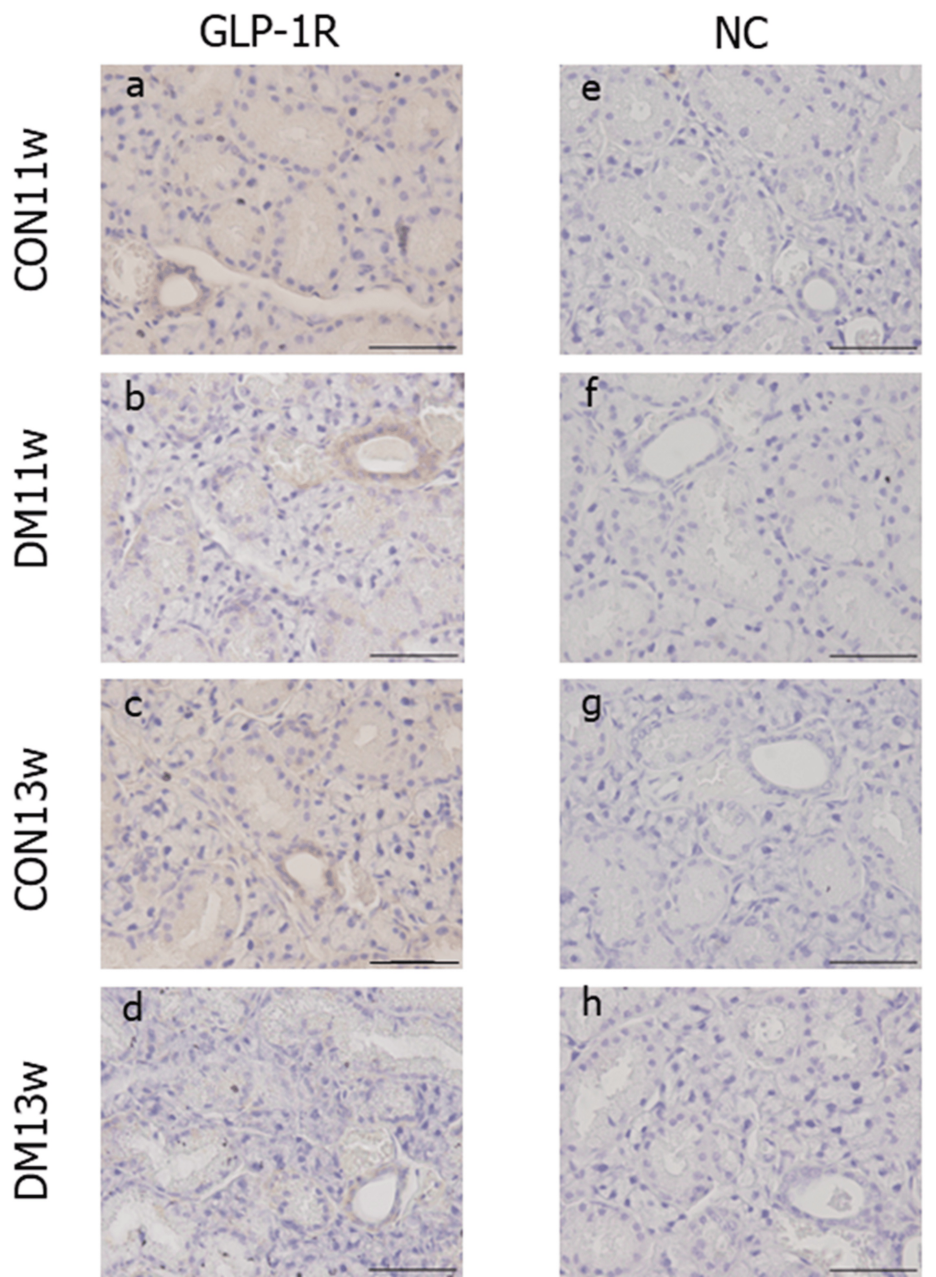
Immunohistochemical images of GLP-1R expression In the CON group, GLP-1R was strongly detected in the duct area, especially in the excretory duct, while not detected in the acinar (a, c). In the DM group, GLP-1R localization was unchanged; expression was weaker in the intercalated duct than in the CON group (b, d). Negative control (e-h) is also shown. The scale bar represents 50 µm. a~d: magnification: 400x CON: control group; DM: diabetes mellitus; NC: negative control; GLP-1R: Glucagon-like peptide-1 receptor

**Figure 9:**
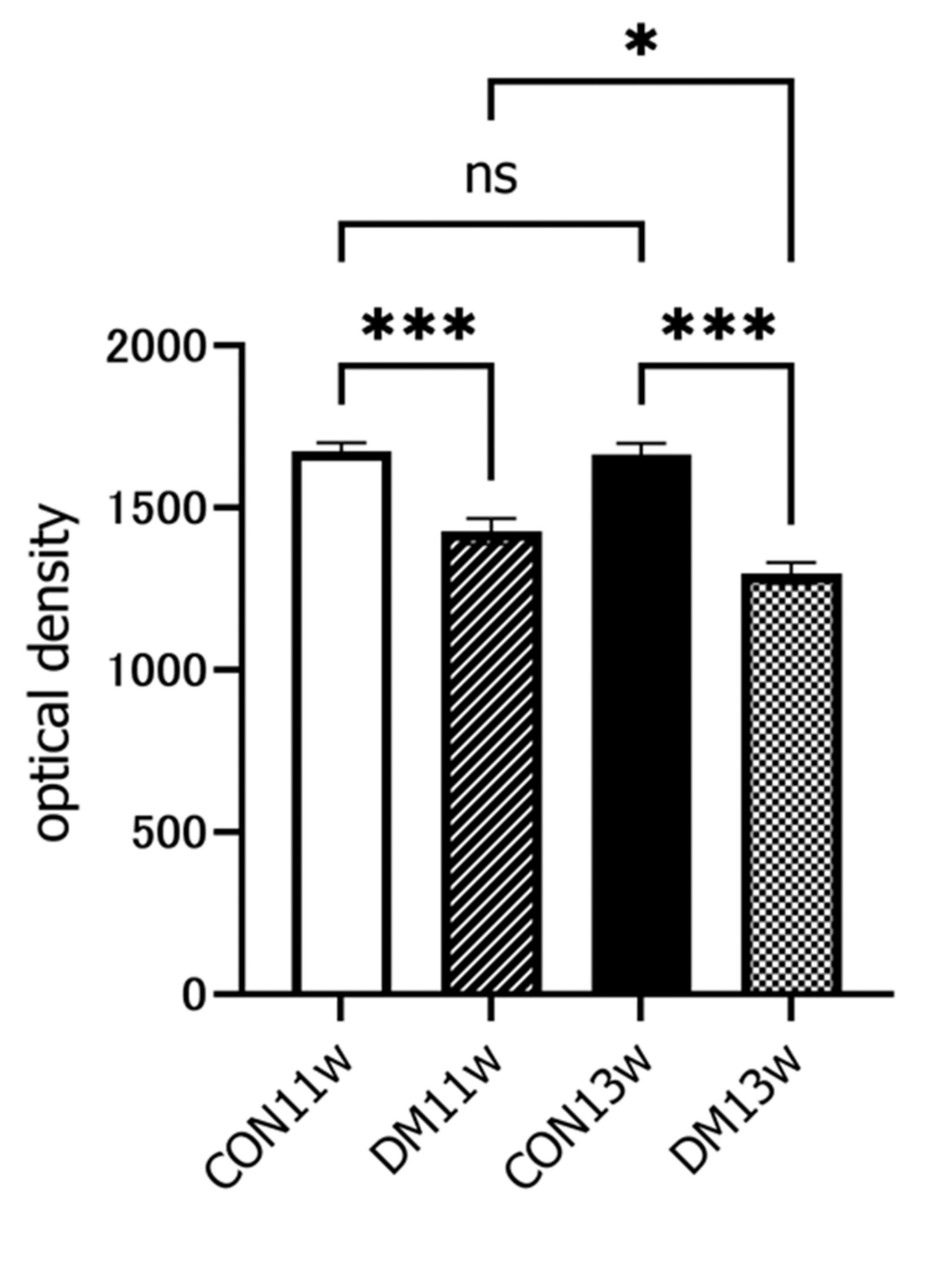
Quantitative analysis of GLP-1R expression in SMGs Anti-GLP-1R immune-positive areas were observed under a light microscope. Two sections per sample and one section per six fields of view of 300 × 300 pixels (0.05 x 0.05 mm) were observed at 400x magnification. Positive immunohistochemical regions were extracted using a digital image analyzer and the immune intensity in each image was measured as optical density. ^*^p<0.05; ^***^p<0.001 CON, control group; DM, diabetes mellitus; ns: not significant; GLP-1R: Glucagon-like peptide-1 receptor; SMG: submandibular gland

The specificity of GLP-1R immunohistochemical staining was demonstrated using negative controls, which did not exhibit nonspecific staining. In normal salivary glands, GLP-1R was not expressed in the acini but was localized in the ducts (striated, intercalated, and excretory ducts), with particularly high intensity in the excretory ducts (Figure 8). In the CON and DM groups, GLP-1R was expressed only in the duct area during all experimental periods. Immunity intensity using OD values in the DM group at both 11 and 13 weeks of age was significantly reduced compared to the respective CON group (Figure 9). Immuno-intensity was also significantly decreased from 11 to 13 weeks of age in the DM group.

## Discussion

This study is the first to observe the impact of T2DM on the expression of salivary gland GLP-1R using the HFD/STZ model mice commonly employed to simulate T2DM pathology. Additionally, by investigating two different periods of T2DM induction, temporal changes were also observed. The HFD/STZ model was chosen because it replicates environmental factors significant in human T2DM more accurately than genetically modified models, which do not precisely reflect the clinical T2DM pathology. This model induces T2DM in mice through a combination of an HFD and low-dose STZ administration. An HFD alone can cause inflammation and oxidative stress in pancreatic β-cells, leading to pancreatic dysfunction and increased insulin resistance in peripheral tissues; however, it is challenging to induce β-cell dysfunction to the extent seen in T2DM. STZ selectively exerts toxicity on pancreatic β-cells and can induce diabetes, but high doses can lead to massive β-cell destruction, resulting in type 1 diabetes pathology. Therefore, low-dose STZ is used to achieve partial β-cell destruction. This combination more closely mimics the environmental factors significant in human T2DM compared to genetically modified models. It has been reported that C57BL/6J mice are prone to obesity from an HFD and exhibit increased blood glucose levels and insulin resistance, making them suitable for creating a T2DM model [15].

Following HFD administration, body weight was significantly increased, but both the DM and CON groups exhibited a decrease at 11 weeks of age and 12 weeks of age following overnight fasting for OGTT. Temporary weight loss and sustained hyperphagia were observed following STZ administration. It has been reported that this is due to a decrease in plasma insulin/leptin levels induced by STZ, leading to reduced signaling of insulin and leptin in the hypothalamus, resulting in the promotion of the orexigenic peptide hypothalamic neuropeptide Y release and inhibition of the anorexigenic peptide alpha-melanocyte-stimulating hormone secretion [16]. Weight loss at 11 weeks is considered due to pre-STZ administration fasting and temporary STZ-induced weight loss, while the weight loss at 12 weeks is likely due to fasting in preparation for OGTT. Weight increase at 13 weeks indicates the mice were not in a starvation state. It has been shown that the AUC at 11 and 13 weeks in the DM group was significantly larger than in the CON group. Thus, a significant deterioration in glucose tolerance and decreased insulin secretion capacity in the DM group at 11 and 13 weeks of age is presumed. Insulin not only lowers postprandial blood glucose levels but also controls lipid metabolism [17]. Insulin secreted in response to meals increases the uptake of lipids into adipose tissue and muscles [18]. The standard diet had an energy content of 3.402 kcal/g with 4.6% of calories from fat, while the HFD had a total energy content of 5.076 kcal/g with 32% of calories from fat, significantly higher than the standard diet. Thus, the mice that developed diabetes through HFD/STZ exhibited worsened lipid metabolism due to decreased insulin secretion capacity.

The impact of T2DM extends to multiple organs, including the oral cavity. The effects of T2DM on salivary glands primarily manifest as symptoms of xerostomia, with established functional and histological findings. However, the mechanisms underlying histological structural changes in T2DM are not yet fully understood. The human SMG accounts for over 70% of total saliva production and is the largest of the three major salivary glands in rodents [19]. Given the significant functional impact and prominent histological changes induced by T2DM, the SMG was the focus of this study. Our results show that the proportion of acinar area increased with aging in both the CON and DM groups at 13 weeks when compared to 11 weeks. While there was no significant difference between the DM11w and CON11w groups, the acinar area increased in the DM13w group compared to the CON13w group. This initial impact of T2DM suggests that atrophic acinar parenchyma is compensated by fibrous tissue replacement [20]. Furthermore, the DM13w group showed a decrease in the proportion of ductal area compared to the CON13w group. Similar findings have been reported in db/db mice, confirming greater changes due to differences in the duration of the study and the earlier onset of diabetes [11]. The expansion of the acinar area and reduction of the ductal area observed in our study are consistent with previous findings. Although changes in the proportion of the ductal area were observed at 13 weeks, there was no significant difference in the number of ductal cells between the groups, suggesting that T2DM induces shrinkage rather than destruction of ductal cells. The enlargement of the ductal lumen observed in this study further supports this result. Xerostomia and decreased salivary secretion are major salivary gland symptoms of T2DM. Xerostomia in T2DM is associated not only with the functional decline of acinar parenchyma but also with the atrophy of ductal cells. Aquaporin-5 (AQP-5), which plays an important role in fluid synthesis and transport, is expressed in the salivary glands. A previous study has shown that T2DM reduces AQP-5 mRNA expression in the SMG [21]. This change in AQP-5 aligns with the decreased salivary flow and the ductal cell atrophy observed in the SMG in our study.

Immunohistochemical examination revealed that GLP-1R in the SMG was distributed exclusively in the ductal region. Consistent with previous studies reporting the expression of GLP-1R in the ductal region of rat major salivary glands, our results align with those findings [4,5]. Salivary glands, like the pancreas, are of endodermal origin, and there have been reports of hormones secreted from the salivary glands, similar to insulin secretion from the pancreas [22]. It has been reported that GLP-1R is expressed not only on pancreatic islet β cells but also on the membranes of pancreatic ducts in mice, rats, as well as humans [23]. The localization of GLP-1R in both the SMG and pancreas being limited to ducts suggests a potential commonality in the function of GLP-1R in SMG and pancreas as part of the same glandular tissue. We observed a decrease in immunostaining-positive areas in the DM group. T2DM is known to cause a decrease in incretin function and impaired GLP-1 secretion in the pancreas, resulting in reduced blood GLP-1 levels [24]. A decrease in immunostaining-positive areas of GLP-1R was observed in the DM group, likely due to decreased incretin function and impaired GLP-1 secretion in the pancreas, affecting peripheral tissues, including the SMG.

The impaired function of the pancreas in T2DM is attributed to increased production of reactive oxygen species (ROS) [25]. Mitochondria, organelles responsible for adenosine triphosphate (ATP) production through aerobic respiration, play a key role in energy generation. ROS are produced as byproducts during ATP production and contribute to oxidative stress. Exposure of mitochondria to high glucose levels increases ROS production, leading to rapid fragmentation [26]. Oxidative stress-induced dysfunction of mitophagy in pancreatic β-cell mitochondria results in impaired insulin secretion [27]. GLP-1 acts on damaged mitochondrial membranes of pancreatic β-cells to facilitate recovery in vitro [28]. Similar effects of T2DM on mitochondria have been observed in skeletal muscle and the brain [29], raising the possibility of similar effects on the salivary glands, which are similar to the pancreas. In other words, under normal conditions, damaged salivary gland ductal cells may be repaired by the action of GLP-1R. However, in T2DM, increased damage to salivary gland ductal cells due to high glucose stress, coupled with a decrease in GLP-1R, may lead to reduced recovery function of SMG ductal cells and ductal cell atrophy. This could be one of the causes of xerostomia in T2DM. However, mechanisms involving the function of GLP-1R in the salivary glands and the impact of T2DM on salivary gland GLP-1R remain unreported. Further research is needed to elucidate the new functions of GLP-1R.

The occlusal state is closely related to oral function. For example, malocclusion due to maxillary molar extraction affects the expression levels of taste receptors on the tongue and AQP-5 in the submandibular gland [30]. Furthermore, regarding submandibular gland GLP-1R, a decrease in immuno-positive areas of GLP-1R in a malocclusion model of rats with extracted teeth has been reported [4], which is similar to the results of decreased immuno-positive areas of GLP-1R observed in this study due to T2DM. Chewing and glucose metabolism are also reported to be related. Chewing has been reported to induce insulin secretion in humans [31], and the digestive activity of chewing may affect postprandial blood glucose control. There is a relationship between occlusion and glucose metabolism, and malocclusion may be a cause of changes in the submandibular gland GLP-1R. However, the relationship and mechanism are still largely unclear. Further exploration of the function of GLP-1R in the submandibular gland is necessary to elucidate the relationship between occlusion and DM.

## Conclusions

T2DM has been shown to induce histopathological changes in SMGs. Our immunohistochemical examination suggests that T2DM may decrease salivary gland GLP-1R expression. These findings provide a new perspective on how salivary glands may be involved in glucose metabolism. Additionally, our finding suggests that GLP-1R in the SMG could be a potential therapeutic target of T2DM in the future. Further research is needed to clarify the relationship between the salivary glands and systemic glucose metabolism. Our findings may be an important first step in elucidating the functional role of salivary glands in T2DM.
